# Trends in evolution of the Rhodniini tribe (Hemiptera, Triatominae): experimental crosses between *Psammolestes tertius* Lent & Jurberg, 1965 and *P. coreodes* Bergroth, 1911 and analysis of the reproductive isolating mechanisms

**DOI:** 10.1186/s13071-021-04854-8

**Published:** 2021-07-02

**Authors:** Amanda Ravazi, Jader de Oliveira, Fabricio Ferreria Campos, Fernanda Fernandez Madeira, Yago Visinho dos Reis, Ana Beatriz Bortolozo de Oliveira, Maria Tercília Vilela de Azeredo-Oliveira, João Aristeu da Rosa, Cleber Galvão, Kaio Cesar Chaboli Alevi

**Affiliations:** 1 Instituto de Biociências, Universidade Estadual Paulista “Júlio de Mesquita Filho” (UNESP), Rua Dr. Antônio Celso Wagner Zanin 250, Distrito de Rubião Júnior, Botucatu, SP 18618-689 Brazil; 2 Laboratório de Entomologia em Saúde Pública, Departamento de Epidemiologia, Faculdade de Saúde Pública, Universidade de São Paulo (USP), Av. Dr. Arnaldo 715, São Paulo, SP Brazil; 3 Laboratório de Parasitologia, Departamento de Ciências Biológicas, Faculdade de Ciências Farmacêuticas, Universidade Estadual Paulista “Júlio de Mesquita Filho” (UNESP), Rod. Araraquara-Jaú km 1, Araraquara, SP 14801-902 Brazil; 4 Laboratório de Biologia Celular, Departamento de Biologia, Instituto de Biociências, Letras e Ciências Exatas, Universidade Estadual Paulista “Júlio de Mesquita Filho” (UNESP), Rua Cristovão Colombo 2265, São José do Rio Preto, SP 15054-000 Brazil; 5 Laboratório Nacional e Internacional de Referência em Taxonomia de Triatomíneos, Instituto Oswaldo Cruz (FIOCRUZ), Av. Brasil 4365, Pavilhão Rocha Lima, sala 505, Rio de Janeiro, RJ 21040-360 Brazil

**Keywords:** Triatomines, Hybridization, *Psammolestes* genus, Hybrid’s unviability, Hybrid collapse

## Abstract

**Background:**

The tribe Rhodniini is a monophyletic group composed of 24 species grouped into two genera: *Rhodnius* and *Psammolestes*. The genus *Psammolestes* includes only three species, namely *P. coreodes*, *P. tertius* and *P. arthuri*. Natural hybridization events have been reported for the Rhodniini tribe (for genus *Rhodnius* specifically). Information obtained from hybridization studies can improve our understanding of the taxonomy and systematics of species. Here we report the results from experimental crosses performed between *P. tertius* and *P. coreodes* and from subsequent analyses of the reproductive and morphological aspects of the hybrids.

**Methods:**

Crossing experiments were conducted between *P. tertius* and *P. coreodes* to evaluate the pre- and post-zygotic barriers between species of the Rhodniini tribe. We also performed cytogenetic analyses of the F1 hybrids, with a focus on the degree of pairing between the homeologous chromosomes, and morphology studies of the male gonads to evaluate the presence of gonadal dysgenesis. Lastly, we analyzed the segregation of phenotypic characteristics.

**Results:**

Interspecific experimental crosses demonstrated intrageneric genomic compatibility since hybrids were produced in both directions. However, these hybrids showed a high mortality rate, suggesting a post-zygotic barrier resulting in hybrid unviability. The F1 hybrids that reached adulthood presented the dominant phenotypic segregation pattern for *P. tertius* in both directions. These insects were then intercrossed; the hybrids were used in the cross between *P. tertius* ♀ × *P. coreodes* ♂ died before oviposition, and the F1 hybrids of *P. coreodes* ♀ x *P. tertius* ♂ oviposited and their F2 hybrids hatched (however, all specimens died after hatching, still in first-generation nymph stage, pointing to a hybrid collapse event). Morphological analyses of male gonads from F1 hybrids showed that they did not have gonadal dysgenesis. Cytogenetic analyses of these triatomines showed that there were metaphases with 100% pairing between homeologous chromosomes and metaphases with pairing errors.

**Conclusion:**

The results of this study demonstrate that *Psammolestes* spp. have intrageneric genomic compatibility and that post-zygotic barriers, namely unviability of hybrid and hybrid collapse, resulted in the breakdown of the hybrids of *P. tertius* and *P. coreodes*, confirming the specific status of species based on the biological concept of species.

**Graphical abstract:**

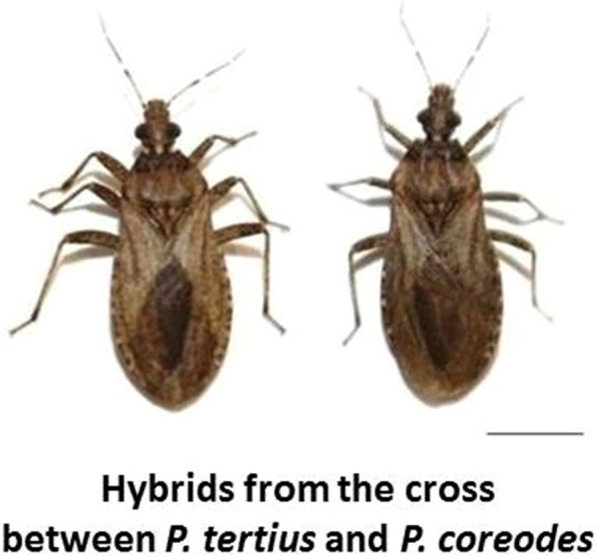

## Background

Chagas disease is a neglected disease which affects about 8 million people worldwide, with approximately 25 million people at risk of infection. Treatment with the anti-trypanosomatids benznidazole and nifurtimox is more effective in the acute phase of the disease than in the chronic phase [1, 2]. Chagas disease is caused by the protozoan *Trypanosoma cruzi* (Chagas, 1909) (Kinetoplastida, Trypanosomatidae) and the main form of transmission is through hematophagous insects known as triatomines [2]. Currently, 156 species of triatomines, divided into 18 genera and five tribes [3–5], have been identified, and all are considered to be potential Chagas disease vectors.

The tribe Rhodniini Pinto, 1926 is a monophyletic group composed of 24 species [3, 5–7] grouped into two genera that are morphologically and ecologically distinct: the members genus genus *Rhodnius* Stål, 1859 have long thin legs and a long head, and live mainly in palm trees, while members of genus *Psammolestes* Bergroth, 1911 have a short head, strong legs, wide femora, a very wide rostrum (the widest in the subfamily) and live in nests of birds of the family Furnariidae [8]. The inclusion of these two genera within the Rhodniini tribe is based on their mainly arboreal behavior and the presence of post-ocular tuberosities [7].

*Rhodnius* is a paraphyletic genus formed by 21 species divided into the trans-Andean *Rhodnius* clade (*pallescens* group) and the cis-Andean *Rhodnius* clade (*pictipes* + *prolixus* groups) [5–7, 9]. The event of paraphilia is supported by the greater evolutionary proximity of the species of the *prolixus* group with the genus *Psammolestes* which groups these species into a single clade [6, 10]. Based on this phenomenon, Hypsa et al. [11] proposed changing the name of the genus including *Psammolestes* spp. to *Rhodnius*: *P. arthuri* (Pinto, 1926) for *R. arthuri* (Pinto, 1926), *P. coreodes* Bergroth, 1911 for *R. coreodes* (Bergroth, 1911) and *P. tertius* Lent & Jurberg, 1965 for *R. tertius* (Lent & Jurberg, 1965).

The distribution of species included in genus *Psammolestes* is restricted to Latin America, with *P. coreodes* being reported in Argentina, Bolivia, Brazil and Paraguay, *P. tertius* in Brazil and Peru and *P. arthuri* in Colombia and Venezuela [12, 13]. Phylogenetic and cytogenetic analyses suggest that this genus is monophyletic [14, 15]. Monteiro et al. [14] suggested that perhaps *Psammolestes* should be regarded as a specialized lineage of *Rhodnius* from the *prolixus* group because the genus *Psammolestes* and species of the *prolixus* group share a common ancestral form, which highlights the paraphyly of the genus *Rhodnius*. Soares et al. [9] also suggested that *Psammolestes* is derived from an ancestral form similar to *R. robustus* Larrousse, 1927, and de Paula et al. [16] suggested that the species *P. coreodes* and *P. tertius* originated by vicariance.

de Paula et al. [16] also suggested that hydrological connections between the Araguaia–Amazon basins in the Early and Middle Miocene [20.4–9.0 million years ago (Mya)] would be a possible event that split the cis-Andean + trans-Andean clades of the *R. domesticus* Neiva & Pinto, 1923 + *Psammolestes* + *prolixus* group (15.2 Mya). These authors indicated that this connection would incorporate the Pantanal system in the Upper Miocene–Lower Miocene (10.0–4.5 Mya), resulting in the separation of the Pantanal system from the Atlantic Forest system, an event that may have contributed to the formation of *Psammolestes* species at a time when *P. coreodes* and *P. tertius* originated (4.98 Mya). On the other hand, Soares et al. [9] suggested that *Psammolestes* spp. spread from the Amazon region northward into the llanos of Venezuela (where *P. arthuri* is abundant in Furnariidae nests) and southeastward into the Caatinga–Cerrado path of Central Brazil (with subsequent differentiation of *P. tertius* along a north–south cline and *P. coreodes* in the Chaco region of Argentina and Paraguay) [14, 17].

Events of natural hybridization have been reported for the Rhodniini tribe (for the genus *Rhodnius* specifically) [18]. Information gained from hybridization studies can further our understanding of the taxonomy and systematics of species, and be used to analyze the isolating mechanisms that limit gene flow between species, and experimental crosses can be employed to establish the role of natural hybridization in generating new genetic variants (that may lead to adaptive evolution and/or in founding new evolutionary lineages) [19, 20]. In this context, we performed, for the first time, experimental crosses between *P. tertius* and *P. coreodes* and analyzed the reproductive and morphological aspects of the hybrids in order to characterize the possible barriers reproductive and the segregation of phenotypic characters, respectively.

## Methods

### Experimental crosses

To evaluate the pre- and post-zygotic barriers between the species of the Rhodniini tribe, we performed crossing experiments between *P. tertius* and *P. coreodes* (Table 1).

**Table 1:** Experimental crosses performed between *P. coreodes* and *P. tertius*

Crossing experiments	Number of eggs				Egg fertility	Mortality F1	Mortality F2
	C1^a^	C2^a^	C3^a^	Total			
*P. coreodes* ♀ × *P. tertius* ♂	3	24	27	54	43%	91.3%	–
*P. tertius* ♀ × *P. coreodes* ♂	40	23	54	117	26%	93.3%	–
Hybrid ♀ × Hybrid ♂^b^	0	–	–	–	–	–	–
Hybrid ♀ × Hybrid ♂^c^	14	–	–	–	57%	–	100%

Control experiments	C1	C2	C3	Total			
*P. coreodes* ♀ × *P. coreodes* ♂	61	10	90	161	68%	–	–
*P. tertius* ♀ × *P. tertius* ♂	52	48	54	154	77%	–	–

^a^ C1, C2 and C3 refer to replicates of experimental crosses

^b^Hybrids of the cross between *P. tertius* ♀ x *P. coreodes* ♂

^c^ Hybrids of the cross between *P. coreodes* ♀ x *P. tertius* ♂

The crossing experiments were conducted in the Triatominae insectary of the School of Pharmaceutical Sciences, São Paulo State University (UNESP), Araraquara, São Paulo, Brazil, according to the experiments described by Mendonça et al. [21] and Neves et al. [22]. The insects were sexed at the fifth-instar nymph stage (N5) [23], and males and females were kept separately until they reached the adult stage in order that only adult virgins were used in the crosses. For the crossing experiments, three couples from each direction were kept in plastic jars [5 (diameter) ×10 cm (height)] and kept at room temperature. Intraspecific crosses were also performed as controls (Table 1). The eggs were collected weekly throughout the females’ oviposition periods, and the egg fertility rate and mortality rate of the hybrids were calculated (Table 1). After the hybrids from the first generation (F1) reached N5, a hybrid pair F1 was formed for each direction (Table 1) and the same parameters described above were used in the evaluation of these crosses.

### Cytogenetic analysis

After the experimental crosses, the F1 males were dissected and the testes removed and stored in a methanol:acetic acid solution (3:1). Slides were prepared by the cell-crushing technique (as described by Alevi et al. [24]), and cytogenetic analyses were performed to characterize spermatogenesis, with emphasis on the degree of pairing between the homeologous chromosomes [21], using the lacto-acetic orcein technique [24, 25]. The slides were examined under a light microscope (Jenamed; Carl Zeiss, Jena, Germany) that was coupled to a digital camera, with a 1000-fold increase; AxioVision LE version 4.8 imaging software (Carl Zeiss) was used for analysis.

### Morphology of the gonads

The morphology of the male gonads of the F1 hybrids was analyzed under a stereomicroscope microscope (model MZ APO; Leica Microsystems GmbH, Wetzlar, Germany) fitted with the Motic Advanced 3.2 Plus Image Analysis System (Motic, Hong Kong) to evaluate the presence of gonadal dysgenesis (which may be uni- or bilateral) [26].

### Segregation of phenotypic characteristics

The head of F1 male hybrids were measured (MZ APO stereomicroscope and Motic Advanced 3.2 Plus Image Analysis System) to analyze the segregation of phenotypic characteristics, based on the main parameter used in the taxonomic key of Lent and Wygodzinsky [8]. *Psammolestes tertius* is characterized by an anteocular region that is 2- to 2.5-fold longer than the post-ocular region, and *P. coreodes* is characterized by an anteocular region that is no longer than twofold the length of the post-ocular region.

## Results and discussion

It is estimated that the ancestors of the Rhodniini and Triatomini Jeannel, 1919 tribes diverged around 48.9–64.4 Mya, at about the time when South America was beginning to separate from Antarctic and Australia during the Lower Tertiary period [27]. It is believed that radiation of the genus *Rhodnius* occurred from the Amazon region and resulted in three main evolutionary lineages: those in the south (Brazilian Cerrado), in the north (Venezuela) and in the northwest (passing through the Andean Cordillera into the Magdalena valley in Colombia) [28]. The colonization of bromeliads, palms trees and bird nests represent important events for the speciation of these taxa [16]. In addition, *Psammolestes* spp. adapted to exploit bird nest microhabitats and currently occur over the open ecoregions north and south of the moist Amazon forests: *P. arthuri* in the Orinoco and Venezuelan coastal basins, *P. tertius* primarily in the Cerrado-Caatinga and *P. coreodes* primarily in the Chaco [14, 29].

**Fig. 1:**
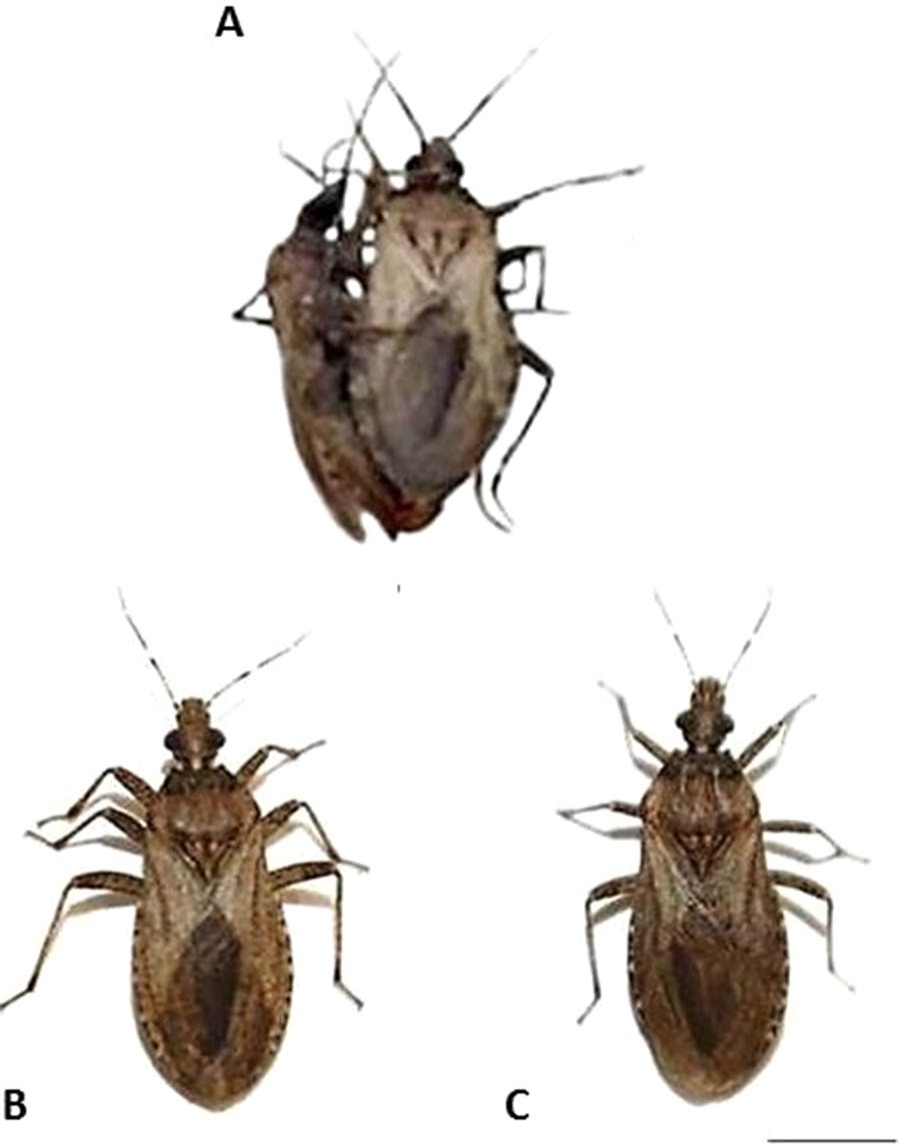
Interspecific experimental crosses between *P.coreodes* and *P. tertius*, and resulting hybrids. **a** Crosses between *P. tertius* ♀ and *P. coreodes* ♂, **b** adult hybrids from the experimental cross between *P. coreodes* ♀ and *P.tertius* ♂, **c** adult hybrids from the experimental cross between *P. tertius* ♀ and *P. coreodes* ♂.* Bar*: 1 cm

Interspecific experimental crosses between *P. tertius* and *P. coreodes* (Fig. 1a) demonstrated intrageneric genomic compatibility since hybrids were produced in both directions (Table 1; Fig. 1b, c). Likewise, interspecific crosses between members of genus *Rhodnius* (*R. prolixus* Stål, 1859 × *R. neglectus* Lent, 1954, *R. prolixus* × *R. robustus*, *R. prolixus* × *R. pictipes* Stål, 1872 and *R. pallescens* Barber, 1932 × *R. colombiensis* Mejia, Galvão & Jurberg, 1999) also resulted in the production of hybrids in at least one of the directions [30–32]. According to the biological concept of species proposed by Mayr [33], i.e. “groups of natural populations that actually or potentially intersect and are reproductively isolated from other groups”, this feature is extremely important from an evolutionary point of view because it demonstrates that evolutionary events resulting in total pre-zygotic isolation between species have not yet been established in the Rhodniini tribe.

The main mechanisms of pre-zygotic reproductive isolation observed in the subfamily Triatominae are ecological isolation and mechanical isolation. The first prevents the formation of hybrids between *Triatoma infestans* (Klug, 1834) and *T. platensis* Neiva, 1913, two species that are phylogenetically related [6] but present in different habits (*T. infestans* is associated with domiciliary regions and feeds on mammalian blood [34]; *T. platensis* is associated with bird nests and feeds preferentially on the blood of birds [35]). The second mechanism is associated with structural incompatibility between male and female genitalia and happens with a certain frequency in only one direction of the crosses, such as, for example, at the crossing of *T. platensis* females with *T. delpontei* Romaña & Abalos, 1947 males [36].

Interspecific genomic compatibility between *P. tertius* and *P. coreodes* was confirmed by the hatching of the F1 (Table 1). However, these hybrids showed a high mortality rate (Table 1), which suggests a post-zygotic barrier resulting in hybrid unviability (confirming the species status of *P. tertius* and *P. coreodes*). Recently, this reproductive barrier was observed for crossings between *T. sordida* (Stål, 1859) and *T. rosai* Alevi et al., 2020 [4], contributing to the description of *T. rosai* by integrative taxonomy.

**Fig. 2:**
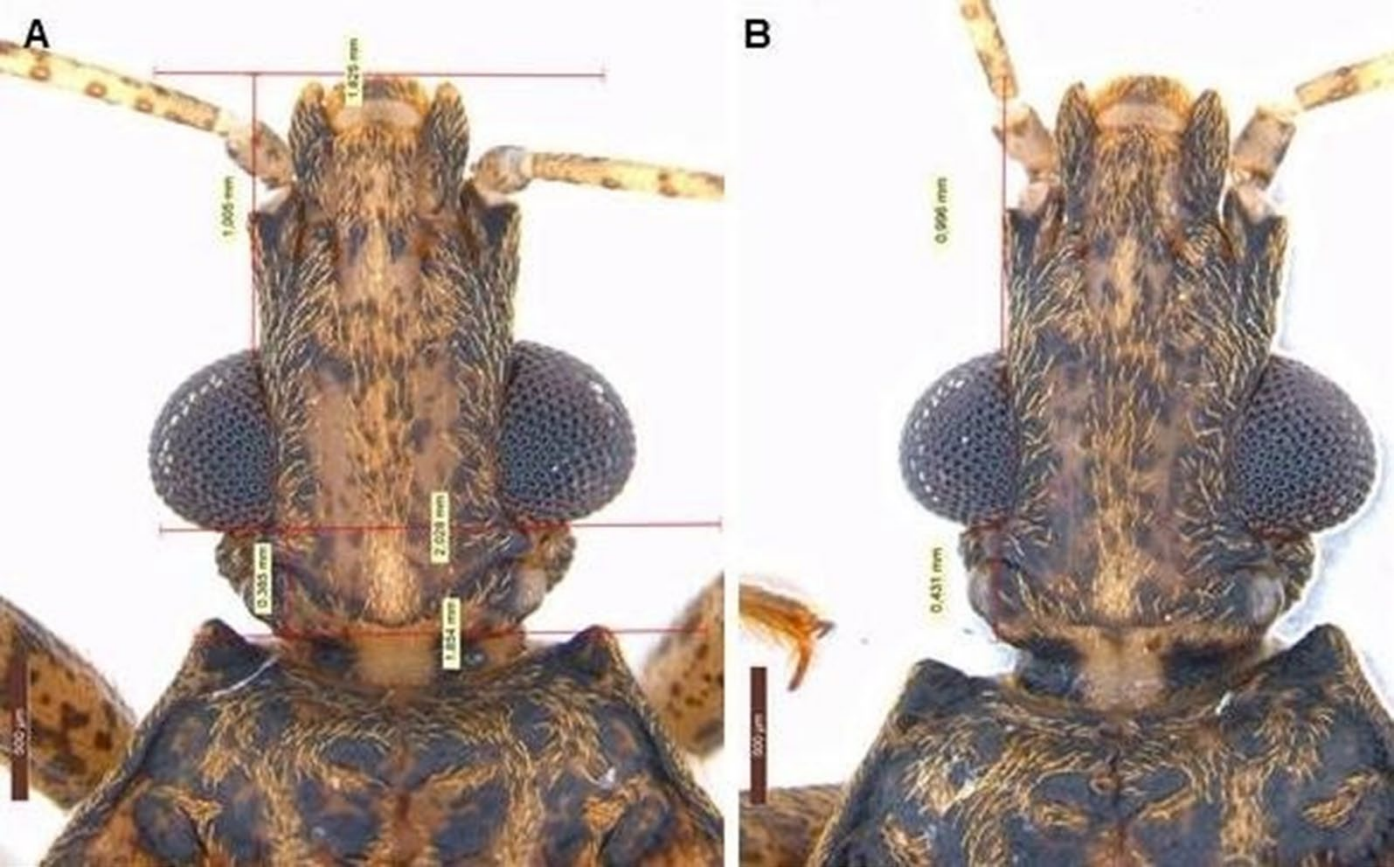
Morphometric analysis of the heads of adult male hybrids of the experimental cross between *P. coreodes* and *P. tertius*. **a** *P. coreodes* ♀ × *P. tertius* ♂ (anteocular region = 1.005 mm; post-ocular region = 0.385 mm; anteocular region is 2.6-fold greater than the post-ocular region), **b** *P. tertius* ♀ × *P. coreodes* ♂ (anteocular region = 0.996 mm; post-ocular region = 0.431; anteocular region is 2.3-fold greater than the post-ocular region)

The few F1 hybrids that reached adulthood presented the dominant phenotypic segregation pattern for *P. tertius* in both directions (anteocular region measuring at least twofold greater than the post-ocular region) (Fig. 2). This is the first study on the segregation of phenotypic characters in the Rhodniini tribe. However, similar analyses have already been carried out in the Triatomini tribe: hybrids of the *T. brasiliensis* complex, for example, presented intermediate characteristics or a specific segregation pattern depending on the crossed species [21, 37, 38]. In addition, Mexican triatomine F1 hybrids also resulted in F1 offspring that were morphologically indistinguishable from one of the parental lines [39] and in second-generation hybrids (F2) with phenotypic characteristics either specific to those of one of the parents or with intermediate characteristics [40, 41]. Considering that some factors can result in an increased risk of *T. cruzi* transmission to humans and animals (such as vigor [42] and hybrid fitness [43]) and that recently it has been reported that hybrids of the *T. phyllosoma* subcomplex show greater potential to acquire and transmit *T. cruzi* than parental species [44, 45], the study of segregation of external morphology can also be of epidemiological importance [37, 38].

The F1 hybrids used in the cross between *P. tertius* ♀ x *P. coreodes* ♂ died before oviposition (confirming the phenomenon of hybrid unviability). This phenomenon has already been characterized for the Triatomini tribe: Martínez-Ibarra et al. [46] observed total mortality of nymphs resulting from crossing species of the *T. phyllosoma* subcomplex with *T. mexicana* (Herrich-Schaeffer, 1848). In addition, Martínez-Ibarra et al. [47–49] observed a lack of hybrid fitness resulting from the crossing between species of the *T. phyllosoma* subcomplex, which resulted in the mortality of nymphs due to not feeding (in the case of the initial stages) and problems during molting (in the older nymphs).

The crossing between the F1 hybrids of *P. coreodes* ♀ × *P. tertius* ♂ oviposited and the F2 hybrids hatched (Table 1). However, all specimens died after hatching, still in first-generation nymph (N1) stage (Table 1). The low adaptive value of hybrids from F2 (which resulted in insect mortality) characterizes the collapse of the hybrid. This phenomenon has already been observed in *Triatoma* Laporte, 1832 hybrids [21, 50] and has been used to confirm the species status of species of the *T. brasiliensis* complex [21, 50].

**Fig. 3:**
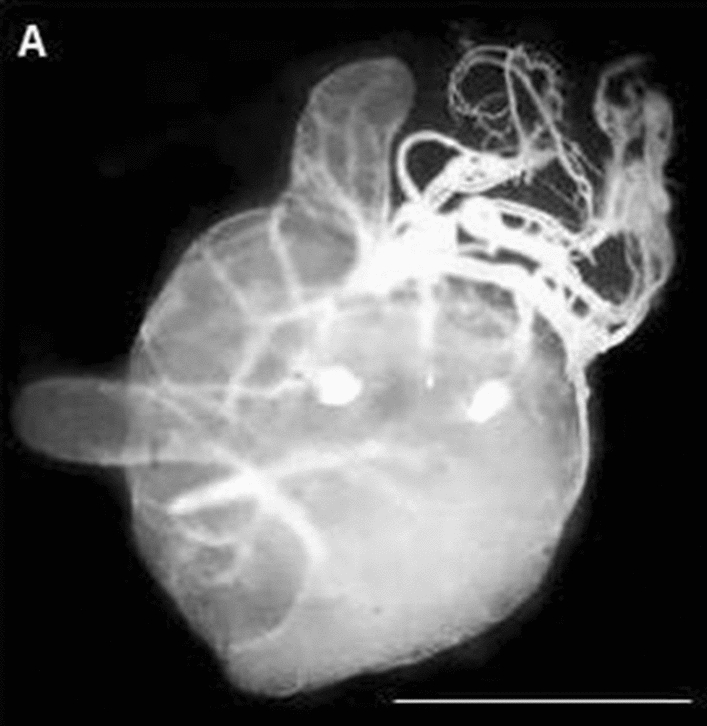
Not atrophied testicle of adult crossbreeding between *P. coreodes* ♀ × *P. tertius* ♂, demonstrating the absence of gonadal dysgenesis at this junction, in both directions.* Bar*: 10 mm

**Fig. 4:**
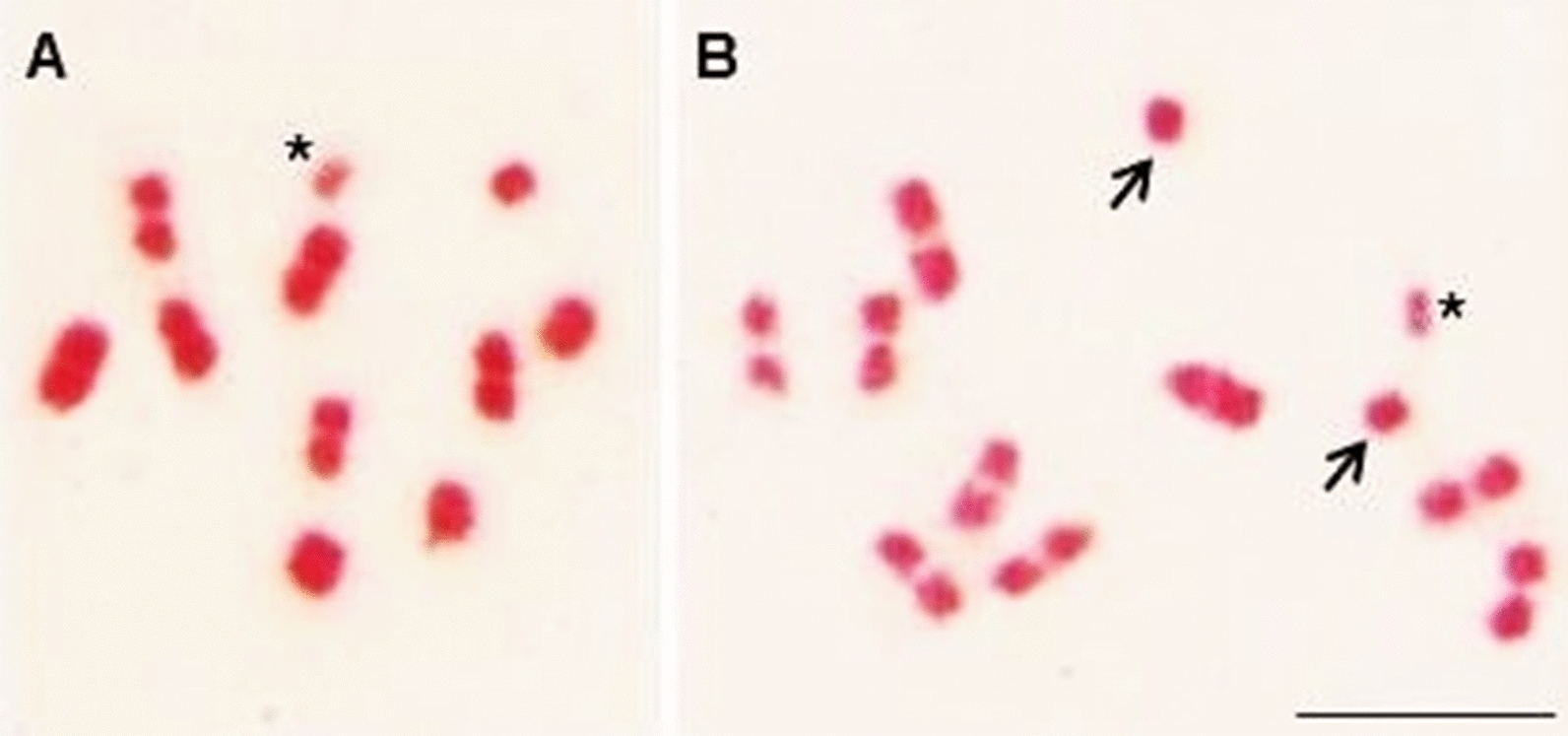
Metaphase during meiosis from the experimental crossing between *P. coreodes* and *P. tertius*. **a** Metaphase with correct pairing of the homeologous chromosomes of *P. coreodes* ♀ × *P. tertius* ♂, **b** metaphase with chromosome pairing error of *P. tertius* ♀ × *P. coreodes* ♂ as indicated by the arrow. Asterisk indicates the sex chromosomes X and Y).* Bar*: 10 μm

Morphological analyses of male gonads from F1 hybrids showed that these hybrids did not have gonadal dysgenesis (Fig. 3). Cytogenetic analyses of these triatomines showed that there were metaphases with 100% pairing between homeologous chromosomes (Fig. 4a) (which justifies hatching of F2 hybrids) and metaphases with pairing errors (Fig. 4b). These results are important from a taxonomic point of view, since according to Riley [51], two species possess distinct genomes when their chromosomes are different in structure and genetic content, so that there is no pairing between one or more pairs of homeologous chromosomes during hybrid meiosis. This behavior leads to sterility and, consequently, to genetic isolation between species.

## Conclusion

The results of our study demonstrated that *Psammolestes* spp. have intrageneric genomic compatibility and that there are post-zygotic barriers (hybrid unviability and hybrid collapse) resulting in the breakdown of the hybrids of *P. tertius* and *P. coreodes*. These results confirm the species status of species based on the biological concept of species.

## Data Availability

The data supporting the conclusions of this article are included within the article.
